# Adolescent Thalamoprefrontal Inhibition Leads to Changes in Intrinsic Prefrontal Network Connectivity

**DOI:** 10.1523/ENEURO.0284-24.2024

**Published:** 2024-08-23

**Authors:** David Petersen, Ricardo Raudales, Ariadna Kim Silva, Christoph Kellendonk, Sarah Canetta

**Affiliations:** ^1^Departments of Psychiatry, Vagelos College of Physicians and Surgeons, Columbia University Irving Medical Center, New York, New York 10032; ^2^Molecular Pharmacology & Therapeutics, Vagelos College of Physicians and Surgeons, Columbia University Irving Medical Center, New York, New York 10032; ^3^Divisions of Molecular Therapeutics, New York State Psychiatric Institute, New York, New York 10032; ^4^Developmental Neuroscience, New York State Psychiatric Institute, New York, New York 10032

**Keywords:** adolescence, excitation inhibition balance, prefrontal cortex maturation, thalamocortical projections

## Abstract

Adolescent inhibition of thalamocortical projections from postnatal days P20 to 50 leads to long-lasting deficits in prefrontal cortex function and cognition in the adult mouse. While this suggests a role of thalamic activity in prefrontal cortex maturation, it is unclear how inhibition of these projections affects prefrontal circuitry during adolescence. Here, we used chemogenetic tools to inhibit thalamoprefrontal projections in male/female mice from P20 to P35 and measured synaptic inputs to prefrontal pyramidal neurons by layer (either II/III or V/VI) and projection target (mediodorsal thalamus (MD), nucleus accumbens (NAc), or callosal prefrontal projections) 24 h later using slice physiology. We found a decrease in the frequency of excitatory and inhibitory currents in layer II/III NAc and layer V/VI MD-projecting neurons while layer V/VI NAc-projecting neurons showed an increase in the amplitude of excitatory and inhibitory currents. Regarding cortical projections, the frequency of inhibitory but not excitatory currents was enhanced in contralateral mPFC-projecting neurons. Notably, despite these complex changes in individual levels of excitation and inhibition, the overall balance between excitation and inhibition in each cell was only altered in the contralateral mPFC projections. This finding suggests homeostatic regulation occurs within subcortically but not intracortical callosal-projecting neurons. Increased inhibition of intraprefrontal connectivity may therefore be particularly important for prefrontal cortex circuit maturation. Finally, we observed cognitive deficits in the adult mouse using this narrowed window of thalamocortical inhibition.

## Significance Statement

Connectivity between two brain regions, the thalamus and the prefrontal cortex, has been found to be reduced in patients with schizophrenia. Neuronal activity in thalamocortical projections is important for the proper development of sensory cortices. How thalamocortical activity regulates prefrontal cortex development is less well understood. Here, we show that decreasing activity in thalamoprefrontal projections in mice during early adolescence alters synaptic connectivity to distinct neuronal projections within the prefrontal cortex that are already evident in adolescence. While some of these changes can be explained by reduced thalamocortical projections, other adaptations are intrinsic to the prefrontal cortex. These findings implicate adolescence as a critical period of cortical development and demonstrate this period as a potential target for therapeutic intervention.

## Introduction

The prefrontal cortex (PFC) supports high level cognitive functioning including working memory and other executive functions (Fuster, 2001; Miller et al., 2018). An impairment in the development or maturation of the prefrontal cortex during adolescence has been postulated to contribute to the cognitive deficits observed in patients with schizophrenia (Feinberg, 1982; Paus et al., 2008; Gogtay et al., 2011; Hoops and Flores, 2017; Larsen and Luna, 2018; Sakurai and Gamo, 2019; Chini and Hanganu-Opatz, 2021; Dienel et al., 2022; Yang and Tseng, 2022). Thalamic inputs regulate sensory cortical maturation during sensitive time windows of development (Wiesel and Hubel, 1963; Nabel and Morishita, 2013). We recently established in the mouse that thalamic input activity during adolescence is necessary for adult prefrontal cortex function and behavior (Benoit et al., 2022). More specifically, we found that decreasing activity in the mediodorsal thalamus (MD) from postnatal day 20–50 using pharmacogenetic tools led to deficits in the performance of two prefrontal-dependent cognitive tasks addressing working memory and attentional set shifting. These deficits were measured in the adult mouse and associated with decreased anatomical thalamocortical projections and decreased functional excitatory postsynaptic currents measured in layer II/III prefrontal pyramidal neurons. Moreover, mice with thalamic inhibition during adolescence showed decreased correlated activity between neurons in the medial prefrontal cortex (mPFC) while performing an attentional set shifting task (ASST) and the ability of the prefrontal cortex to decode task outcomes was impaired (Benoit et al., 2022). Notably, decreasing the activity of thalamoprefrontal projections for a comparable length period in adulthood did not lead to the same long-lasting effects.

**Table 1.** *N* and statistical significance for all electrophysiology experiments

		mCherry		hM4D		hM4D + NaCl		All statistical tests are linear mixed effects models			
		A.	C.	A.	C.	A.	C.		Amplitude	Median Inst. Freq.	Frequency
L II/III	**N**	**8**	**42**	**7**	**28**	**5**	**24**	**E**	(*F*_(2,18.71)_ = 2.414; *p* = 0.117)	**(*F*_(2,90)_ = 15.553, *p* < 0.001)↓**	**(*F*_(1,92)_ = 12.271; *p* < 0.001)↓**
	♂	2		5		3		**I**	(*F*_(2,16.30)_ = 0.269; *p* = 0.768)	(*F*_(2,17.03)_ = 0.785; *p* = 0.472)	(*F*_(2,16.39)_ = 1.141; *p* = 0.344)
	♀	6		2		2		**E/I**	(*F*_(2,5.47)_ = 1.966; *p* = 0.173)	(*F*_(2,17.63)_ = 0.770; *p* = 0.478)	(*F*_(2,16.55)_ = 1.196; *p* = 0.327)
II/III→NAc.	N	**5**	**21**	**6**	**28**	–		**E**	(*F*_(1,6.19)_ = 3.313; *p* = 0.117)	(*F*_(1,6.77)_ = 1.926; *p* = 0.208)	**(*F*_(1,47)_ = 9.354; *p* = 0.004)↓**
	♂	4		4				**I**	(*F*_(1,7.03)_ = 3.402; *p* = 0.107)	(*F*_(1,7.79)_ = 0.955; *p* = 0.358)	**(*F*_(1,4.89)_ = 9.451; *p* = 0.028)↓**
	♀	1		2				**E/I**	(*F*_(1,6.59)_ = 0.543; *p* = 0.487)	(*F*_(1,7.91)_ = 0.836; *p* = 0.388)	(*F*_(1,8.10)_ = 0.750; *p* = 0.411)
V/VI→MD	N	**4**	**19**	**5**	**26**	–		**E**	(*F*_(1,5.36)_ = 0.689; *p* = 0.442)	**(*F*_(1,6.10)_ = 13.045; *p* = 0.011)↓**	**(*F*_(1,6.73)_ = 12.960; *p* = 0.009)↓**
	♂	1		3				**I**	(*F*_(1,4.77)_ = 0.991; *p* = 0.367)	**(*F*_(1,5.96)_ = 6.501; *p* = 0.044)↓**	**(*F*_(1,6.39)_ = 7.829; *p* = 0.029)↓**
	♀	3		2				**E/I**	(*F*_(1,42)_ = 3.625; *p* = 0.064)	(*F*_(1,5.21)_ = 0.975; *p* = 0.367)	(*F*_(1,5.31)_ = 1.115; *p* = 0.337)
V/VI→NAc.	N	**5**	**22**	**4**	**9**	–		**E**	**(*F*_(1,29)_ = 8.976; *p* = 0.006)↑**	(*F*_(1,6.24)_ = 0.196; *p* = 0.673)	(*F*_(1,5.49)_ ≤ 0.001; *p* = 0.993)
	♂	4		2				**I**	**(*F*_(1,4.25)_ = 14.809; *p* = 0.013)↑**	(*F*_(1,6.53)_ = 0.910; *p* = 0.374)	(*F*_(1,6.82)_ = 0.417; *p* = 0.539)
	♀	1		2				**E/I**	(*F*_(1,28)_ = 2.220; *p* = 0.147)	(*F*_(1,12.33)_ = 0.412; *p* = 0.533)	(*F*_(1,12.10)_ = 0.014; *p* = 0.908)
II/III→II/III	N	**5**	**38**	**5**	**26**	–		**E**	(*F*_(1,6.37)_ = 2.865; *p* = 0.134)	(*F*_(1,7.30)_ = 0.399; *p* = 0.547)	(*F*_(1,7.41)_ = 0.012; *p* = 0.917)
	♂	2		2				**I**	(*F*_(1,62)_ = 0.112; *p* = 0.912)	**(*F*_(1,5.95)_ = 8.023; *p* = 0.030)↑**	**(*F*_(1,7.32)_ = 4.185; *p* = 0.078)↑**
	♀	3		3				**E/I**	(*F*_(1,59)_ = 1.099; *p* = 0.299)	**(*F*_(1,9.34)_ = 5.744; *p* = 0.039)↓**	**(*F*_(1,11.37)_ = 5.697; *p* = 0.035)↓**

A, animal; C, cell; all significant results are bolded and italicized with directionality indicated by an arrow.

These findings established that thalamoprefrontal activity is required during adolescence to sustain thalamocortical connectivity, prefrontal network function, and cognitive behavior in adulthood. However, it was not clear whether these effects on circuit connectivity were apparent immediately after the adolescent manipulation or if they require maturation into adulthood to manifest. Moreover, how adolescent thalamocortical inhibition impacts populations of mPFC pyramidal neurons differentiated by both cortical layer and projection target remained unknown. The latter question is important for understanding the scope of this manipulation's impact on the intrinsic connectivity of the prefrontal cortex.

To address these questions, we undertook a detailed study of prefrontal-cortical connectivity using slice electrophysiology 24 h following pharmacogenetic inhibition of thalamoprefrontal projections from postnatal days 20 to 35. We chose this reduced window of inhibition because prior work demonstrated changes in the density of thalamic projections to the mPFC following adolescent thalamic inhibition were already present at this time (Benoit et al., 2022). We first recorded excitatory and inhibitory currents from layer II/III neurons as previously done in the adult (Benoit et al., 2022) to establish whether the observed deficit in excitation arises during adolescence. We then restricted our recordings to specific mPFC-projecting neurons identified by their projection target (the MD, nucleus accumbens (NAc), or callosal mPFC) and cortical layer. We chose mPFC- and MD-projecting neurons as they are largely distinguished by cortical layer (II/III vs V/VI, respectively) and NAc-projecting neurons as they span both layers and therefore provide a within-layer comparison for the other two populations. We found excitatory postsynaptic currents are reduced in layer II/III nonprojection-specific neurons during adolescence, as observed in adult mice. When analyzing specific projections, we found a reduction in frequency of excitatory and inhibitory currents to layer V/VI corticothalamic neurons and layer II/III cortico-accumbens–projecting neurons while layer V/VI cortico-accumbens–projecting neurons displayed increased amplitudes of excitatory and inhibitory currents. While we observed changes in excitation or inhibition in subcortically projecting neurons, the excitation/inhibition balance was not affected, suggesting homeostatic mechanisms may offset decreased excitatory inputs. In contrast, callosal corticocortical projection neurons displayed a significant decrease in excitatory/inhibitory (E/I) balance. Together, these data show a complex adaptation of cortical networks in response to adolescent thalamocortical inhibition and suggest that activity in callosal-projecting neurons in the mPFC may be most profoundly affected due to a lack of homeostatic adaptation.

To determine whether this narrowed window (P20–P35) of adolescent thalamocortical inhibition would impact cognitive flexibility in the adult mouse, we performed an attentional set shifting behavioral task. Like P20–50 inhibition (Benoit et al., 2022), inhibition from P20 to P35 was sufficient to induced long-lasting deficits in cognition.

## Materials and Methods

### Animal husbandry

All procedures were performed in accordance with guidelines approved by the Institutional Animal Care and Use Committees at Columbia University and the New York State Psychiatric Institute (protocol NYSPI 1589). Animals were housed under a 12 h light/dark cycle in a temperature-controlled environment (22°C; humidity of 30–70%) with food and water available *ad libitum*, unless otherwise noted. C57/BL6 females and males (Jackson Laboratories, 000664) were used for all experiments. Littermates were randomly assigned to each experimental group, with random distribution across males and females. Mice were housed together with dams and littermates. Offspring were weaned at P28 and group housed with same-sex littermates (no more than five mice per cage). For thalamic inhibition, mice were given intraperitoneal (i.p.) injections of JHU37160 (J60, a CNO analog) dissolved in 0.9% sterile saline at 0.1 mg/kg twice per day (Bonaventura et al., 2019). J60 was used as an alternative to the widely used clozapine-*N*-oxide (CNO) because J60 is known to have less off-target effects. New evidence has shown CNO might be reverse metabolized into clozapine, a common antipsychotic medication with high affinity to dopamine D2 and serotonin 2A receptors (Manvich et al., 2018). All mice were given J60 regardless of viral vector, except those given saline with hM4D expression. Surgeries were conducted at P12–13, and all mice were injected from P20 to P35. For electrophysiological experiments, 24 h after the final J60 injection, mice were anesthetized with isoflurane and decapitated for slice electrophysiology at P35 (±4 d). For behavioral experiments, mice were tested in an ASST beginning in adulthood (>P90). Throughout data collection and analysis, experimenters were blinded to the group of the animal. We based sample sizes on previous experiments, and no statistical methods were used to calculate sample sizes.

### Surgical procedures

For viral injections at P13, mice were anesthetized with isoflurane and head-fixed in a stereotactic apparatus (Kopf). Mice were injected bilaterally in the midline thalamus with AAV5-hSyn-DIO-hM4D-mCherry (Addgene, 44362; titer, 2.5 × 1,013 vg/ml) or a control virus, AAV5-hSyn-DIO-mCherry (Addgene, 50459; titer, 7 × 10^12^ vg/ml), at a volume of 0.25 µl (0.1 µl/min). Mice were also injected bilaterally in the mPFC with retrograde AAV-hSyn-Cre-WPRE-hGH (Addgene, 105553; titer, 7 × 10^12^ vg/ml) at a volume of 0.2 µl (0.1 µl/min). For the tracer experiments, mice were injected with retrograde AAV-hSyn-EGFP (Addgene, 50465; titer, 7 × 10^12^ vg/ml) at a volume of 0.25 µl for the MD, 0.2 µl for the mPFC, and 0.1 µl for the NAc. The following were the P13 coordinates: MD thalamus, −0.85 anterior–posterior (AP), ±0.20 medial–lateral (ML), and −3.25 dorsal–ventral (DV, zero at brain surface); mPFC, 2.4 AP, ±0.2 ML and −1.9 DV (zero at brain surface); NAc, 1.7 AP, ±1.1 ML and −5.07 DV. All coordinates were zeroed at bregma unless otherwise noted. Histology images were collected from each animal for each cohort, with at least six slices taken for each animal to determine viral spread and confirm targeting.

### Slice electrophysiology

Whole-cell voltage-clamp recordings were performed in layer II/III mPFC pyramidal neurons and layer V/VI mPFC pyramidal neurons. Recordings were obtained with a MultiClamp 700B amplifier (Molecular Devices) and digitized using a Digidata 1440B acquisition system (Molecular Devices) with Clampex 10 (Molecular Devices) and analyzed with pClamp 10 (Molecular Devices). Following decapitation, 300 µm slices containing the mPFC were incubated in artificial cerebral spinal fluid containing the following (in mM): 126 NaCl, 2.5 KCl, 2.0 MgCl_2_, 1.25 NaH_2_PO_4_, 2.0 CaCl_2_, 26.2 NaHCO_3_, and 10 mM d-glucose, pH 7.45 (310 mOsm) bubbled with oxygen at 32°C for 30 min before being returned to room temperature for at least 1 h before use. During recording, slices were perfused in room temperature artificial cerebral spinal fluid at a rate of 5 ml/min. Electrodes were pulled from 1.5 mm borosilicate glass pipettes on a P-97 puller (Sutter Instruments). Electrode resistance was typically 1.5–3 MΩ when filled with internal solution consisting of the following (in mM): 130 cesium hydroxide monohydrate, 130 mM d-gluconic acid, 10 HEPES, 2.0 MgCL_2_, 0.2 EGTA, 2.5 Mg-ATP, 0.3 Na-GTP, and 5 lidocaine N-ethyl bromide, pH 7.3 (277 mOsm).

#### mPFC recordings

**Figure 1.**
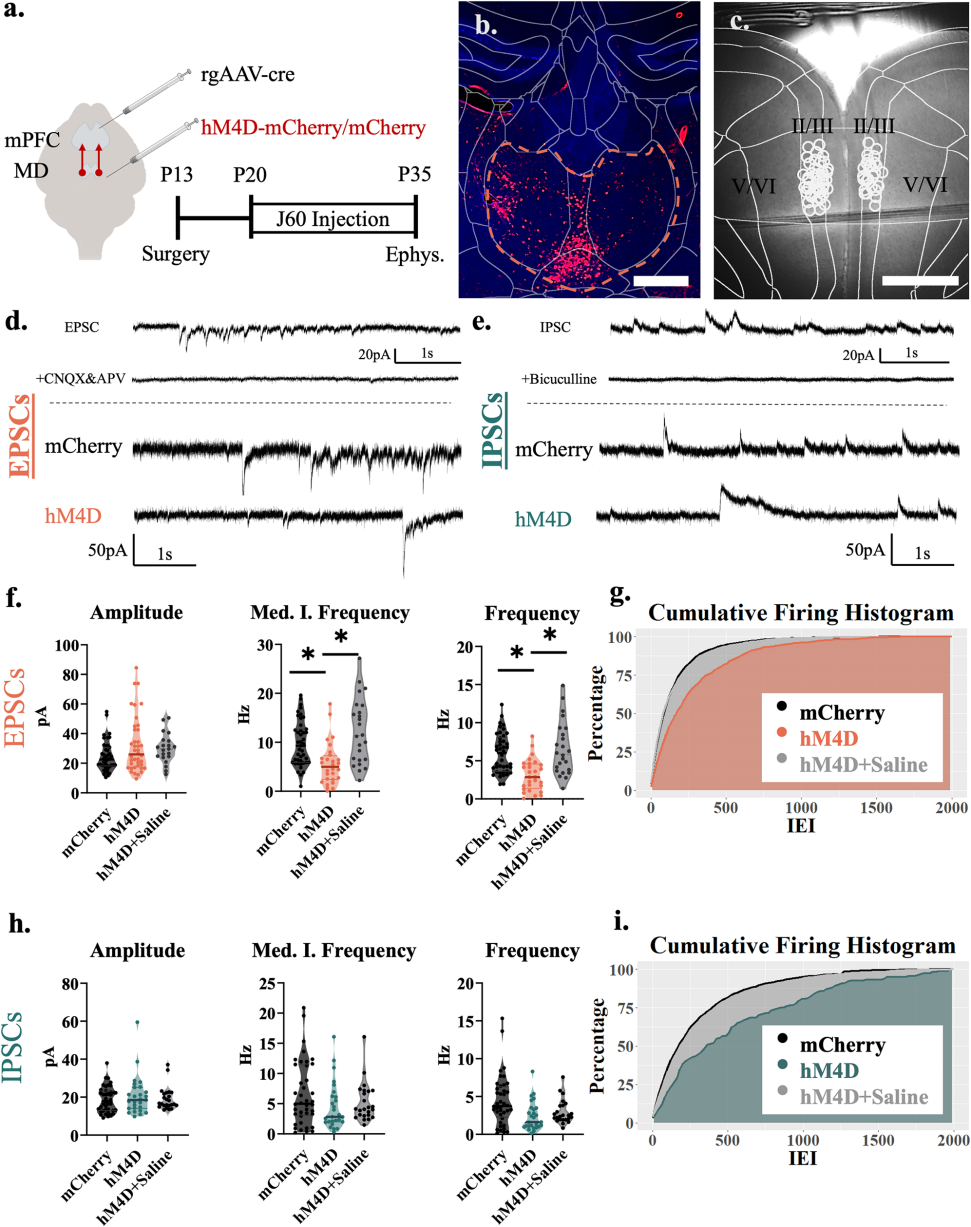
Adolescent thalamocortical inhibition decreases excitatory transmission to pyramidal cells in layer II/III of the mPFC. ***a***, Adolescent experimental timeline and schematic. Whole-cell patch-clamp recordings were made from pyramidal cells in layer II/III of the mPFC from hM4D and control mice. These pyramidal cells receive excitatory inputs from the thalamus as well as inhibitory inputs from local interneurons; created with https://biorender.com. ***b***, Example image illustrating hM4D-mCherry expression in the mediodorsal thalamus (orange outline) in adolescent animals. Histology images were collected from each animal for each cohort, with at least six slices taken for each animal. Scale bar, 200 µm. ***c***, Example image illustrating the recording location in the prelimbic (PrL) subregion of the medial prefrontal cortex. All recordings for this experiment were conducted in this region ∼200–350 µm from the pial surface (L II/III). Each circle represents the approximate location of a recorded cell. Scale bar, 500 µm. ***d***, Top, Baseline EPSC activity at P35, all events cease after application of AMPA and NMDA receptor antagonists, CNQX and APV. Bottom, Representative traces showing sEPSCs of hM4D animals and mCherry controls. ***e***, Top, Baseline IPSC activity at P35, all events cease after application of GABA receptor antagonist bicuculline. Bottom, Representative traces showing sIPSCs of HM4D animals and mCherry controls. ***f***, sEPSC frequency (right) and median instantaneous frequency (middle) are significantly reduced following adolescent thalamic inhibition relative to control mice, but sEPSC amplitude (left) is unchanged; mCherry control, *n* = 42 cells and 8 animals; hM4D, *n* = 28 cells and 7 animals; HM4D + Saline *n* = 24 cells and 5 animals; frequency, mCherry control 5.750(4.183) Hz, hM4D 2.867(2.900) Hz, and hM4D + Saline 5.875(4.862) Hz, contrasts of estimated marginal means revealed a significant difference between hM4D animals and both mCherry, *z* = 4.188, df = *∞* *p_bonferroni_ < 0.001, and HM4D + Saline, *z* = 4.515 df = *∞* *p_bonferroni_ < 0.001 but no significant difference between mCherry and HM4D + Saline, *z* = −1.005, df = *∞* *p_bonferron_*i* = 0.945; median instantaneous frequency, mCherry control 9.292(6.699) Hz, hM4D 4.472(4.044) Hz, hM4D + Saline 11.349(9.957) Hz; contrasts of estimated marginal means revealed a significant difference between hM4D animals and both mCherry, *z* = 4.142, df = *∞* *p_bonferroni_ < 0.001, and HM4D + Saline, *z* = 5.392, df = *∞* *p_bonferroni_ < 0.001 but no significant difference between mCherry and HM4D + Saline, *z* = −1.953, df = *∞* *p_bonferron_*i* = 0.153; amplitude, mCherry control 21.691(10.399) pA, hM4D 22.066(14.875) pA, and HM4D + Saline 29.665(10.517) pA. All reported values are median(interquartile range). ***g***, Graph of the cumulative firing distribution for sEPSCs in L II/III pyramidal cells in the mPFC; IEI represents the interevent interval between spontaneous events. ***h***, sIPSC amplitude (left), median instantaneous frequency (middle), and frequency (right) are unchanged following adolescent thalamic inhibition relative to control mice; mCherry control, *n* = 42 cells and 8 animals; hM4D, *n* = 28 cells and 7 animals; HM4D + Saline *n* = 24 cells and 5 animals; amplitude, mCherry control 18.503(9.833) pA, hM4D 18.526(9.687) pA, and hM4D + Saline 16.586(6.272); median instantaneous frequency, mCherry control 4.994(8.177) Hz, hM4D 2.829(4.170) Hz, and hM4D + Saline 4.107(3.993); frequency, mCherry control 3.158(3.867) Hz, hM4D 1.433(2.017) Hz and HM4D + Saline 2.425(1.825). All reported values are median(interquartile range). ***i***, Graph of the cumulative firing distribution for sIPSCs in L II/III pyramidal cells in the mPFC; IEI represents the interevent interval between spontaneous events. *p < 0.05.

Animals were killed for recordings at P35 after the adolescent manipulation. mPFC pyramidal neurons were visually identified based on their shape and prominent apical dendrite at ×40 magnification under infrared and diffusion interference contrast microscopy. Recordings were taken from either layer II/III of the mPFC (prelimbic, PrL) or layer V/VI. We focused on the PrL rather than the infralimbic (IL) region as lesions of this region disrupt performance in the ASST we are employing (Bissonette et al., 2008). For the same reason, we also recorded from the prelimbic cortex in our previous study (Benoit et al., 2022). Layer II/III was defined as any cell lying 200–350 µm from the pial surface. Layer V/VI was defined as neurons within 400–600 µm from the pial surface. Spontaneous excitatory postsynaptic currents (sEPSCs) were recorded in voltage clamp at a holding potential of −65 mV, and spontaneous inhibitory postsynaptic currents (sIPSCs) were recorded in voltage clamp at a holding potential of +10 mV. The combination of the intracellular solution and the selected holding potentials allowed us to isolate EPSCs and IPSCs from one another without the use of blockers, which we verified by demonstrating that sEPSCs were completely blocked by administration of 50 µM APV [(2*R*)-amino-5-phosphonovaleric acid] and 20 µM CNQX (6-cyano-7-nitroquinoxaline-2,3-dione), but not 20 µM bicuculline, and vice versa for sIPSCs (Fig. 1*d*). Sixty seconds of the current recording for each condition were analyzed. Recordings were filtered with a 2 kHz eight-pole low-pass Bessel filter, and sEPSCs and sIPSCs were detected using MiniAnalysis (Synaptosoft). Frequency, amplitude, and instantaneous frequency were calculated for all traces. Frequency was calculated by dividing the total number of events by time (60 s). Amplitude was the distance from baseline to the tip of the event. Instantaneous frequency was the inverse of the interevent interval converted to a frequency. All event data were averaged by cell.

### Attentional set shifting behavioral task

At P90, mice were gradually restricted to 85% of their body weight. Mice were habituated to the testing arena on Day 1. On Days 2–3, they were trained to dig in both bedding media (corn cob and paper pellet, both unscented) to obtain a food reward. Once mice dug reliably, testing began. For each trial, mice were placed at the opposite end from two terra cotta bowls containing different odor/medium combinations. For IA, mice needed to learn that the cinnamon scent, not the paprika scent, predicted a Honey Nut Cheerios reward, irrespective of the bedding medium. For the first five trials, mice could explore both bowls until they found the reward, but the trial was only scored as correct if the animal initially chose the correct bowl. From the sixth trial onward, once the mouse began digging in a bowl, the entrance to the other bowl was closed off. The criterion was reached when the mouse made 8 of 10 consecutive correct choices. If the mouse did not meet the criterion in 30 trials, the animal did not advance to the next stage. If the mouse did reach the criterion, the extradimensional set shifting (EDSS) portion of the task began. In EDSS, the animal needed to learn that the type of bedding medium (paper pellets, not corn cobs) predicted the Honey Nut Cheerios reward irrespective of odor. The criterion was reached when the mouse made 8 of 10 consecutive correct choices. All behavioral tasks were conducted during the light cycle.

### Statistics

Statistical analysis and graph preparations were done using Prism 9 software (GraphPad Software), JASP [JASP Team (2023), JASP (Version 0.17.3)], or custom scripts in R. Linear mixed effects models with individual animal used as a random-effects grouping factor were used to analyze slice physiology. Linear mixed effects models were used over a common ANOVA or t test to account for the possible nonindependence of measurements from neurons from the same animal. Prior to analysis, all datasets were tested for outliers, and they were removed using the following equation (Q1–1.5 × IQR or Q3 + 1.5 × IQR), which identifies datapoints ±2.698σ from the mean. For the nonspecific L II/III experiment, two outliers were removed from the EPSC hM4D + Saline amplitude group, one outlier was removed from the IPSC hM4D frequency group, and one outlier was removed from the IPSC hM4D instantaneous frequency group. For the cortico-accumbens experiment, two outliers were removed from the IPSC hM4D group. For the corticocortical population experiment, two outliers were removed from the IPSC mCherry frequency group. For the E/I balance analysis, two outliers were removed from the hM4D corticocortical frequency group. An independent samples t test was used to analyze behavior. Although we analyzed male and female mice in this study, the study was not designed to study sex differences as we were already analyzing range of different cell populations and variables. Sample size and details regarding the statistical analysis for each experiment is summarized in Table 1.

## Results

### P20–P35 thalamocortical inhibition decreases excitatory transmission to pyramidal neurons in layer II/III of the mPFC

We first performed patch-clamp recordings in brain slices from prelimbic layer II/III neurons, which are the mPFC population of projection neurons that obtain the most prominent input from the MD (Anastasiades and Carter, 2021). To inhibit thalamic activity during adolescence on P12–13, we injected an adeno-associated virus (AAV) carrying a Cre-dependent version of the inhibitory designer receptor hM4D into the thalamus of C57 mice and a retrograde virus carrying Cre recombinase, rgAAV-CRE, into the mPFC. Retrograde transport will cause Cre recombinase expression in the MD, leading to hM4D expression selectively in medial prefrontal-projecting neurons (Fig. 1*a*). We then chronically inhibited these cortically projecting MD neurons with the hM4D ligand JHU37160 (J60; 0.1 mg/kg) from P20 until the day before they were recorded (P35 ± 4 d; Fig. 1*b*). To control for the possible nonligand-dependent effects of the hM4D receptor, we also included a group of mice expressing hM4D that were injected with saline during the same developmental window. Twenty-four hours after the last J60 injection (at P35 ± 4 d), we recorded layer II/III neurons and analyzed excitatory and inhibitory postsynaptic currents.

Using linear mixed effects models, we found that inhibition of thalamocortical projections from P20 to P35 decreased the frequency (*F*_(2,92)_ = 12.271; *p* < 0.001) and median of the instantaneous frequency (*F*_(2,90)_ = 15.553; *p* < 0.001) of sEPSCs, while there was no change in the amplitude (*F*_(2,18.71)_ = 2.414; *p* = 0.117) of sEPSCs when compared with controls (Fig. 1*f–i*). Measuring sIPSCs revealed that frequency (*F*_(2,16.39)_ = 1.141; *p* = 0.344), the median of the instantaneous frequency (*F*_(2,17.03)_ = 0.785; *p* = 0.472), and amplitude (*F*_(2,16.30)_ = 0.269; *p* = 0.768) remained unchanged when compared with controls (Fig. 1*f–i*). Furthermore, when comparing the mCherry and Saline groups in a post hoc contrast, no significant differences were observed, signaling the lack of nonligand-dependent effects of the hM4D receptor (EPSC frequency [*z*(*∞*) = 1.005; *p*_bonferroni_ = 0.945]; EPSC median instantaneous frequency [*z*(*∞*) = 1.953; *p*_bonferroni_ = 0.153]) and no overall effect in EPSC amplitude, IPSC frequency, IPSC instantaneous frequency, or IPSC amplitude. These results indicate that adolescent thalamocortical activity causes significant alterations to excitatory transmission in the superficial regions of the mPFC, consistent with what we previously found in adult mice (Benoit et al., 2022). Moreover, the change in frequency, but not amplitude, is consistent with a presynaptic, as opposed to postsynaptic, effect.

### P20–35 thalamocortical inhibition decreases excitatory and inhibitory transmission to layer II/III NAc-projecting neurons in the mPFC

**Figure 2.**
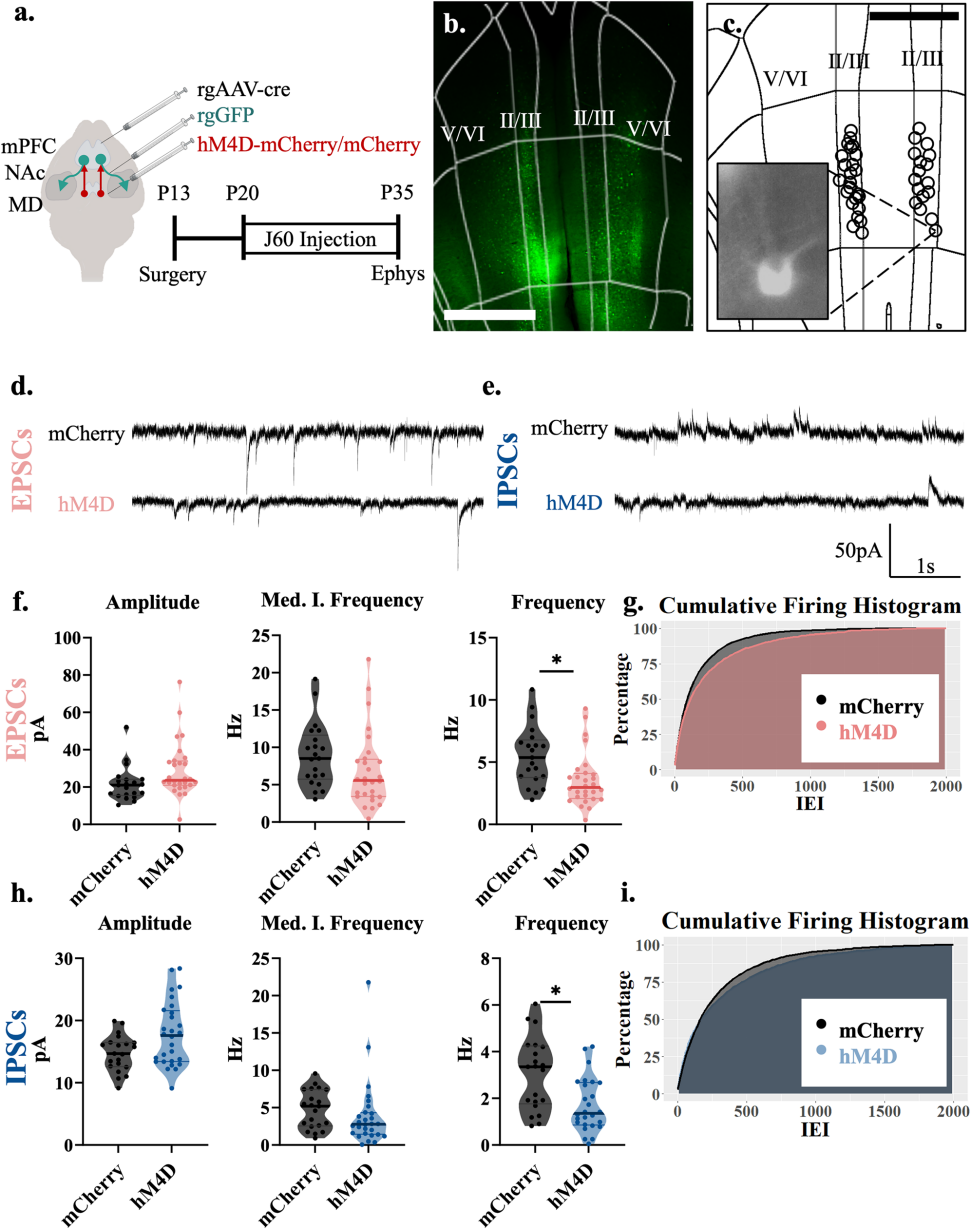
Adolescent thalamocortical inhibition decreases excitatory and inhibitory transmission to layer II/III NAc-projecting cells in the mPFC. ***a***, Adolescent experimental timeline and schematic. Whole-cell patch-clamp recordings were made from accumbens-projecting pyramidal neurons in layer II/III of the mPFC from hM4D and control mice; created with https://biorender.com. ***b***, Example image illustrating rGFP-positive, accumbens-projecting cells in the prelimbic subregion of the medial prefrontal cortex. ***c***, Each circle represents the approximate location of a recorded cell. Scale bar, 500 µm. ***d***, Representative traces showing sEPSCs of hM4D animals and mCherry controls. ***e***, Representative traces showing sIPSCs of hM4D animals and mCherry controls. ***f***, sEPSC frequency (right) is significantly reduced in accumbens-projecting cortical neurons following adolescent thalamic inhibition relative to control mice, but sEPSC amplitude (left) and median instantaneous frequency (middle) are unchanged; mCherry control, *n* = 21 cells and 5 animals; hM4D, *n* = 28 cells and 6 animals; frequency: mCherry control 5.367(2.717) Hz, hM4D 2.975(1.871) Hz; median instantaneous frequency, mCherry control 8.514(4.842) Hz and hM4D 5.579(4.803) Hz; amplitude: mCherry control 21.051(7.992) pA and hM4D 23.648(12.495) pA. All reported values are median(interquartile range). ***g***, Graph of the cumulative firing distribution for sEPSCs in L II/III accumbens-projecting pyramidal cells in the mPFC; IEI represents the interevent interval between spontaneous events. ***h***, sIPSC frequency (right) is significantly reduced in accumbens-projecting cortical cells following adolescent thalamic inhibition relative to control mice, but sIPSC amplitude (left) and median instantaneous frequency (middle) are unchanged; mCherry control, *n* = 21 cells and 5 animals; hM4D, *n* = 28 cells and 6 animals; frequency, mCherry control 3.350(2.350) Hz, hM4D 1.417(1.762) Hz; median instantaneous frequency, mCherry control 5.195(4.817) Hz and hM4D 2.776(2.753) Hz; amplitude, mCherry control 14.618(3.768) pA and hM4D 17.597(7.919) pA. All reported values are median(interquartile range). ***i***, Graph of the cumulative firing distribution for sIPSCs in L II/III accumbens-projecting pyramidal cells in the mPFC. *p < 0.05.

We then determined whether the observed effects of adolescent thalamocortical inhibition includes the layer II/III cortico-accumbens cell populations. To this end, we injected the rgGFP virus into the nucleus accumbens (NAc; Fig. 2*a*,*b*) and recorded from the superficial layers (II/III) of the mPFC (Fig. 2*c–e*). We found that in superficial NAc-projecting neurons, excitatory and inhibitory transmissions were both dampened following adolescent thalamocortical inhibition. For excitatory currents, the frequency (*F*_(1,47)_ = 9.354; *p* = 0.004) was significantly decreased but not the median of the instantaneous frequency (*F*_(1,6.77)_ = 1.926; *p* = 0.208) or the amplitude (*F*_(1,6.19)_ = 3.313; *p* = 0.117; Fig. 2*f,g*). For inhibitory transmission in superficial layers, sIPSC frequency was reduced (*F*_(1,4.89)_ = 9.451; *p* = 0.028) but not the median of the instantaneous frequency (*F*_(1,7.79)_ = 0.955; *p* = 0.358) or the amplitude (*F*_(1,7.03)_ = 3.402; *p* = 0.107; Fig. 2*h,i*). These data show that excitatory and inhibitory inputs are decreased onto layer II/III accumbens-projecting neurons.

### P20–35 thalamocortical inhibition decreases excitatory and inhibitory transmission to MD-projecting neurons in layer V/VI of the mPFC

**Figure 3.**
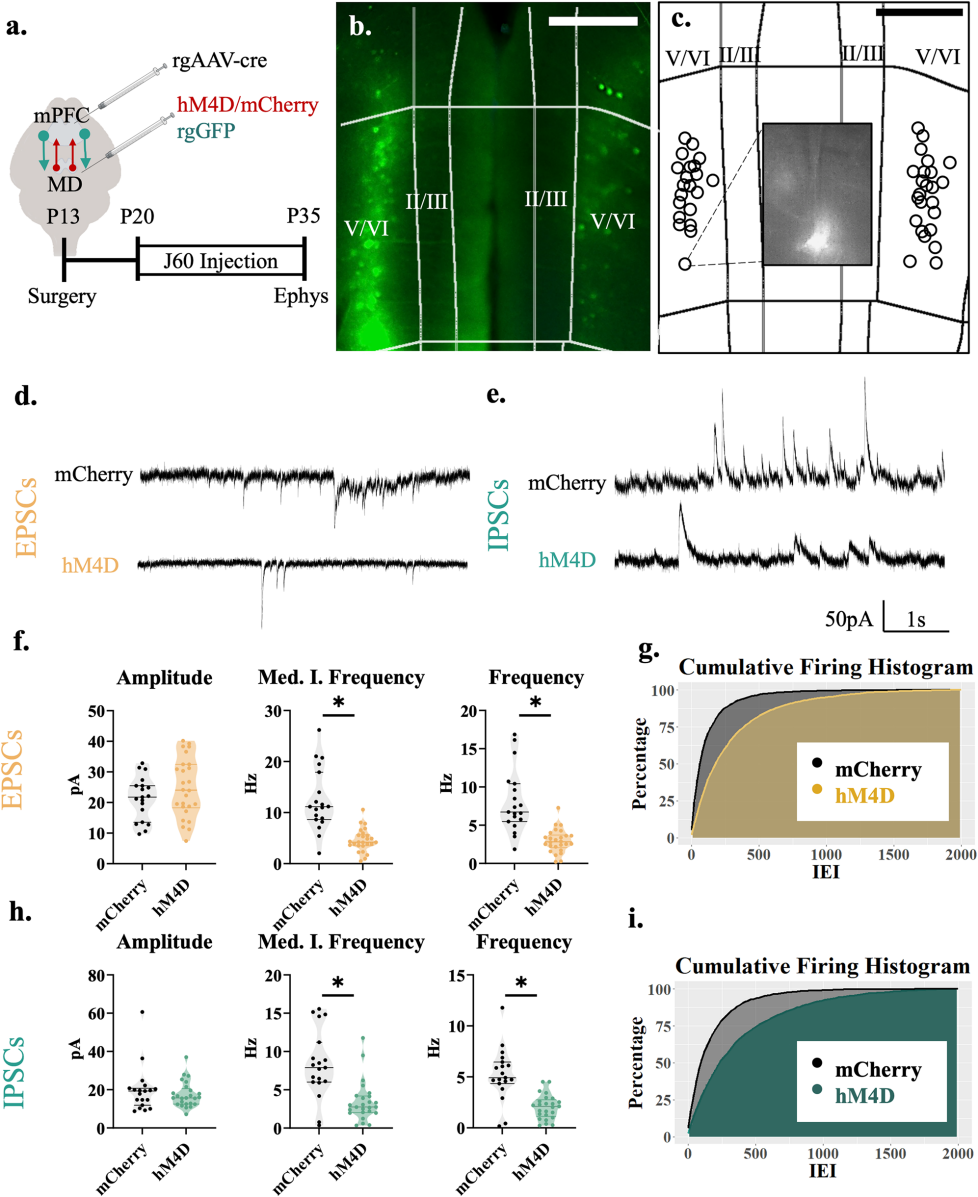
Adolescent thalamocortical inhibition decreases excitatory and inhibitory transmission to MD-projecting cells in layer V/VI of the mPFC. ***a***, Adolescent experimental timeline and schematic. Whole-cell patch-clamp recordings were made from cortical-projecting pyramidal cells in layer V/VI of the mPFC from hM4D and control mice. These pyramidal cells receive excitatory inputs from the thalamus and superficial layers of the cortex as well as inhibitory inputs from local interneurons; created with https://biorender.com. ***b***, Example image illustrating rGFP-positive cells in the prelimbic subregion of the medial prefrontal cortex. All recordings for this experiment were conducted in this region ∼500–600 µm from the pial surface (L V/VI). cR Each circle represents the approximate location of a recorded cell. Scale bar, 300 µm. ***d***, Representative traces showing sEPSCs of hM4D animals and mCherry controls. ***e***, Representative traces showing sIPSCs of hM4D animals and mCherry controls. ***f***, sEPSC frequency (right) and median instantaneous frequency (middle) are significantly reduced in thalamic-projecting cortical cells following adolescent thalamic inhibition relative to control mice, but sEPSC amplitude (left) is unchanged; mCherry control, *n* = 19 cells and 4 animals; hM4D, *n* = 26 cells and 5 animals; frequency, mCherry control 6.717(4.392) Hz and hM4D 2.833(1.408) Hz; median instantaneous frequency, mCherry control 11.173(7.187) Hz and hM4D 4.204(1.997) Hz; amplitude, mCherry control 21.764(9.897) pA and hM4D 24.043(13.508) pA. All reported values are median(interquartile range). ***g***, Graph of the cumulative firing distribution for sEPSCs in L V/VI thalamic-projecting pyramidal cells in the mPFC; IEI represents the interevent interval between spontaneous events. ***h***, sIPSC frequency (right) and median instantaneous frequency (middle) is significantly reduced in thalamic-projecting cortical neurons following adolescent thalamic inhibition relative to control mice, but sIPSC amplitude (left) is unchanged; mCherry control, *n* = 19 neurons and 4 animals; hM4D, *n* = 26 neurons and 5 animals; frequency: mCherry control 4.917(1.825) Hz and hM4D 2.100(1.350) Hz; median instantaneous frequency: mCherry control 7.896(4.100) Hz and hM4D 2.755(2.081) Hz; amplitude: mCherry control 19.267(7.264) pA and hM4D 15.720(7.127) pA. All reported values are median(interquartile range). ***i***, Graph of the cumulative firing distribution for sIPSCs in L V/VI thalamic-projecting pyramidal neurons in the mPFC. *p < 0.05.

We then determined the effects of chronic thalamocortical inhibition on a different subcortical projection, cortical neurons from deep layers V/VI projecting to the MD thalamus. A rgGFP virus was injected into the MD and whole-cell patch-clamp recordings were taken in thalamic-projecting cortical neurons (Fig. 3*a–e*). Like for nonspecific layer II/III and cortico-accumbens neurons, we observed significant decreases in excitatory synaptic transmission (Fig. 3*f,g*). Significant decreases were observed in both the frequency (*F*_(1,6.73)_ = 12.960; *p* = 0.009) and median of the instantaneous frequency (*F*_(1,6.10)_ = 13.045; *p* = 0.011) of sEPSCs but the amplitude (*F*_(1,5.36)_ = 0.689; *p* = 0.442) of sEPSCs remained unchanged (Fig. 3*f,g*). In addition, the frequency (*F*_(1,6.39)_ = 7.829; *p* = 0.029) and median of the instantaneous frequency (*F*_(1,5.96)_ = 6.501; *p* = 0.044) but not the amplitude (*F*_(1,4.77)_ = 0.991; *p* = 0.367) of sIPSCs was reduced after P20–35 thalamic inhibition (Fig. 3*h,i*). These data show that, like for superficial layer II/III accumbens projections, adolescent thalamoprefrontal inhibition also decreases excitatory and inhibitory synaptic inputs to deep layer corticothalamic neurons.

### P20–35 thalamocortical inhibition increases the amplitude of excitatory and inhibitory currents to layer V/VI NAc-projecting neurons in the mPFC

**Figure 4.**
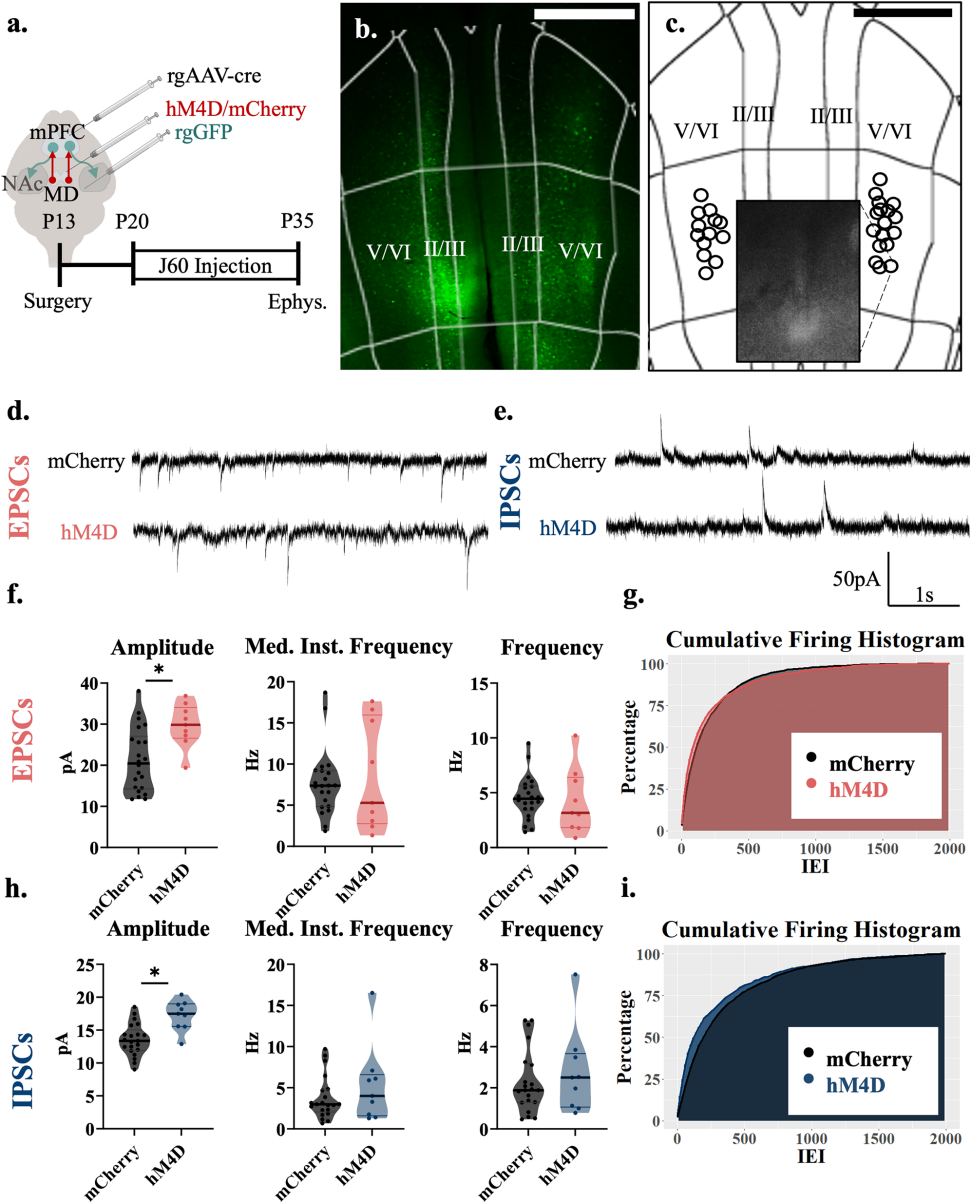
Adolescent thalamocortical inhibition increases the amplitude of excitatory and inhibitory currents to layer V/VI NAc-projecting cells in the mPFC. ***a***, Adolescent experimental timeline and schematic. Whole-cell patch-clamp recordings were made from accumbens-projecting pyramidal neurons in layer V/VI of the mPFC from hM4D and control mice; created with https://biorender.com. ***b***, Example image illustrating rGFP-positive, accumbens-projecting cells in the prelimbic subregion of the medial prefrontal cortex. ***c***, Each circle represents the approximate location of a recorded cell. Scale bar, 500 µm. ***d***, Representative traces showing sEPSCs of hM4D animals and mCherry controls. ***e***, Representative traces showing sIPSCs of hM4D animals and mCherry controls. ***f***, sEPSC amplitude (left) is significantly increased in accumbens-projecting cortical neurons following adolescent thalamic inhibition relative to control mice, but sEPSC frequency (right) and median instantaneous frequency (middle) are unchanged; mCherry control, *n* = 22 neurons and 5 animals; hM4D, *n* = 9 neurons and 4 animals; frequency, mCherry control 4.450(2.012) Hz, hM4D 3.167(4.217) Hz; median instantaneous frequency, mCherry control 7.371(4.043) Hz and hM4D 5.290(12.190) Hz; amplitude, mCherry control 20.460(11.638) pA and hM4D 29.858(5.869). All reported values are median(interquartile range). ***g***, Graph of the cumulative firing distribution for sEPSCs in L II/III accumbens-projecting pyramidal neurons in the mPFC; IEI represents the interevent interval between spontaneous events. ***h***, sIPSC frequency (right) is significantly reduced in accumbens-projecting cortical neurons following adolescent thalamic inhibition relative to control mice, but sIPSC amplitude (left) and median instantaneous frequency (middle) are unchanged; mCherry control, *n* = 21 neurons and 5 animals; hM4D, *n* = 28 neurons and 6 animals; frequency, mCherry control 1.883(1.817) Hz, hM4D 2.50(2.350) Hz; median instantaneous frequency, mCherry control 2.997(2.405) Hz and hM4D 4.005(4.349) Hz; amplitude, mCherry control 13.362(2.306) pA and hM4D 17.495(3.331). All reported values are median(interquartile range). ***i***, Graph of the cumulative firing distribution for sIPSCs in L V/VI accumbens-projecting pyramidal neurons in the mPFC. *p < 0.05.

Accumbens-projecting prefrontal neurons are located in both superficial and deep cortical layers (Anastasiades and Carter, 2021). We therefore also recorded accumbens-projecting neurons in layer V/VI (Fig. 4*a–e*). We found significant increases to excitatory and inhibitory current amplitude and no changes in frequency or median of the instantaneous frequency, in deep layer accumbens-projecting neurons (Fig. 4*f,g*). When measuring excitatory currents in deep layers, amplitude was significantly increased (*F*_(1,29)_ = 8.976; *p* = 0.006); however, frequency (*F*_(1,5.49)_ = 7.841 × 10^−5^; *p* = 0.993) and median of the instantaneous frequency (*F*_(1,6.24)_ = 0.196; *p* = 0.673) remained unchanged (Fig. 4*f,g*). Similar to excitatory transmission, for inhibitory currents in deeper layers, amplitude was significantly increased (*F*_(1,4.25)_ = 14.809: *p* = 0.013); however, frequency (*F*_(1,6.82)_ = 0.417; *p* = 0.539) and median of the instantaneous frequency (*F*_(1,6.53)_ =0.910; *p* = 0.374) remained unchanged (Fig. 4*h,i*). Thus, unlike layer II/III accumbens projection neurons, excitatory and inhibitory drive onto layer V/VI accumbens neurons is not decreased but it is enhanced although at the level of amplitude and not frequency.

### P20–35 thalamocortical inhibition increases inhibitory transmission to layer II/III callosal-projecting neurons in the mPFC

**Figure 5.**
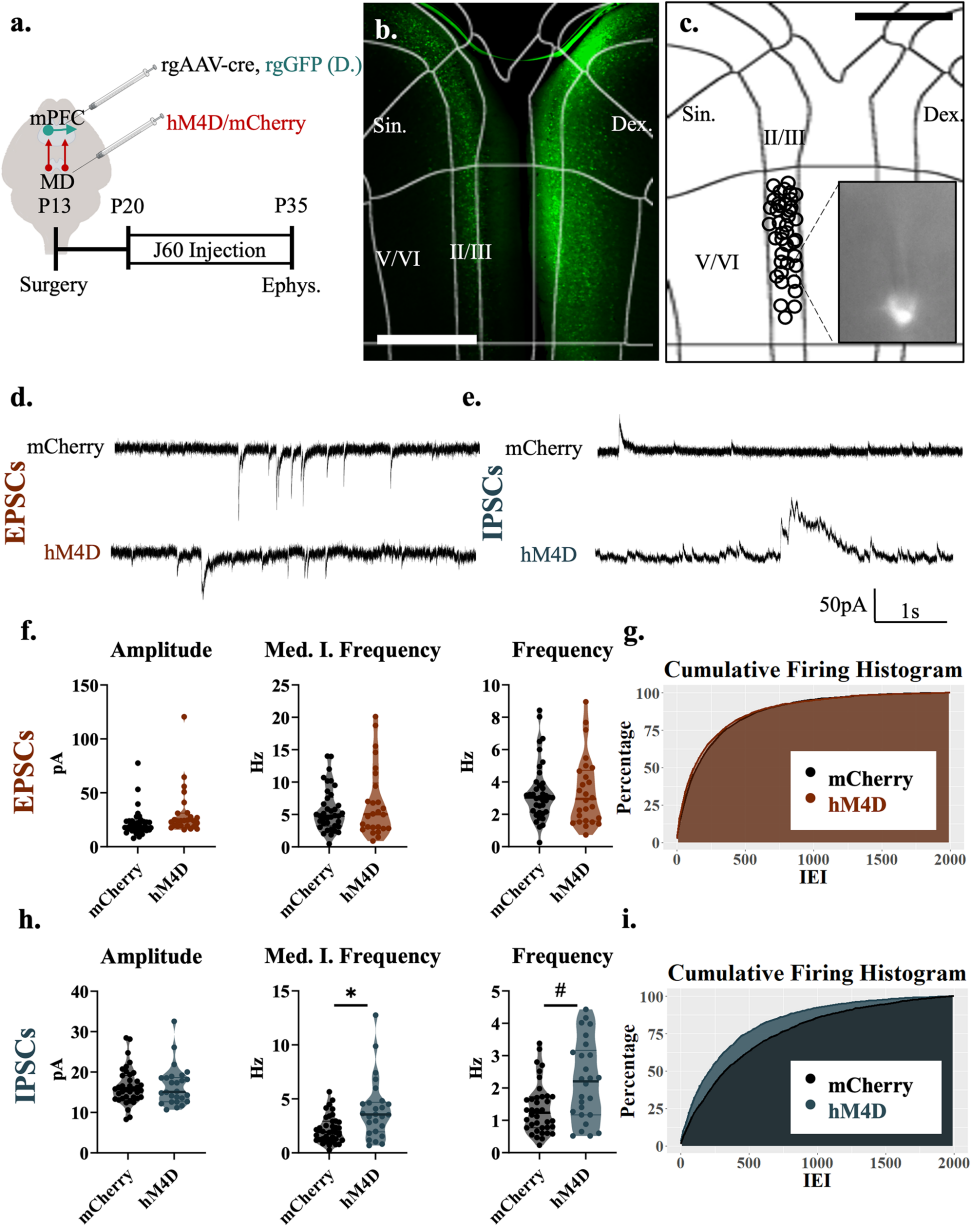
Adolescent thalamocortical inhibition increases inhibitory transmission in layer II/III callosal-projecting cells in the mPFC. ***a***, Adolescent experimental timeline and schematic. Whole-cell patch-clamp recordings were made from cortical-projecting pyramidal neurons in layer II/III of the mPFC from hM4D and control mice; created with https://biorender.com. ***b***, Example image illustrating rGFP-positive, cortical-projecting cells in the prelimbic subregion of the medial prefrontal cortex. Virus was injected into the dextral (right) hemisphere and cells were recorded only in the sinistral (left) hemisphere. ***c***, Each circle represents the approximate location of a recorded cell. Scale bar, 500 µm. ***d***, Representative traces showing sEPSCs of hM4D animals and mCherry controls. ***e***, Representative traces showing sIPSCs of hM4D animals and mCherry controls. ***f***, sEPSC frequency (right), sEPSC amplitude (left), and sEPSC median instantaneous frequency are unchanged in cortical-projecting cortical neurons following adolescent thalamic inhibition relative to control mice; mCherry control, *n* = 38 neurons and 5 animals; hM4D, *n* = 26 neurons and 5 animals; frequency, mCherry control 3.042(1.713) Hz and hM4D 2.95(2.933) Hz; median instantaneous frequency, mCherry control 4.807(3.944) Hz and hM4D 5.042(5.890) Hz; amplitude, mCherry control 17.876(7.549) pA and hM4D 23.951(8.397) pA. All reported values are Median(Interquartile Range). ***g***, Graph of the cumulative firing distribution for sEPSCs in cortical-projecting pyramidal neurons in the mPFC; IEI represents the interevent interval between spontaneous events. ***h***, sIPSC median instantaneous frequency (middle) is significantly increased and frequency (right) is trending toward an increase in cortical-projecting cortical neurons following adolescent thalamic inhibition relative to control mice, while amplitude (left) is unchanged; mCherry control, *n* = 38 neurons and 5 animals; hM4D, *n* = 26 neurons and 5 animals; frequency, mCherry control 1.233(0.954) Hz and hM4D 2.208(1.887) Hz; median instantaneous frequency, mCherry control 1.937(1.676) Hz and hM4D 5.551(2.498); amplitude, mCherry control 15.735(5.268) pA and hM4D 15.099(5.667) pA. All reported values are median(interquartile range). ***i***, Graph of the cumulative firing distribution for sIPSCs in cortical-projecting pyramidal neurons in the mPFC. *p < 0.05, ^#^*p* < 0.10.

To determine how adolescent thalamocortical inhibition impacts corticocortical circuitry, a rgGFP virus was injected into the dextral prelimbic mPFC, and whole-cell patch-clamp recordings were taken in callosal-projecting cortical neurons in the sinistral hemisphere (Fig. 5*a–e*). No significant differences were observed in the frequency (*F*_(1,7.41)_ = 0.012; *p* = 0.917), median of the instantaneous frequency (*F*_(1,7.30)_ = 0.399; *p* = 0.547), or amplitude (*F*_(1,6.37)_ = 2.865; *p* = 0.134) of excitatory events in cortical-projecting cortical neurons (Fig. 5*f,g*). Unlike excitatory transmission, inhibitory transmission significantly increased in the distribution of events (instantaneous frequency; *F*_(1,5.95)_ = 8.023; *p* = 0.030) and trended in frequency (*F*_(1,7.32)_ = 4.185; *p* = 0.078) but not amplitude (*F*_(1,62)_ = 0.112; *p* = 0.912) after adolescent thalamocortical inhibition (Fig. 5*h,i*). These findings were unexpected since callosal-projecting prelimbic neurons are enriched in layer II/III (Anastasiades et al., 2018) and the nonselective analysis of layer II/III neurons displayed a decrease in the frequency of excitatory currents and no increase in inhibition.

### P20–30 thalamic inhibition only alters excitation–inhibition balance in layer II/III callosal-projecting neurons in the mPFC

**Figure 6.**
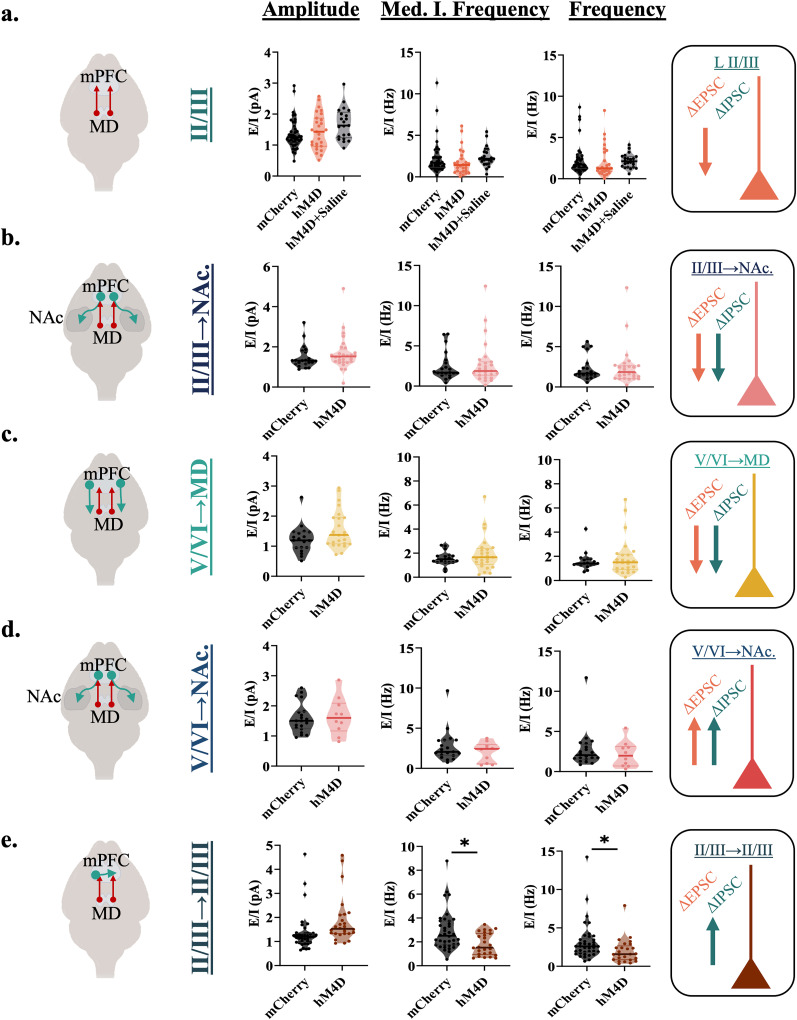
Adolescent thalamic inhibition only alters Excitation-Inhibition balance in layer II/III callosally projecting cells in the mPFC. ***a***, Left panel, Schematic of viral injections. Center panel, Excitatory/inhibitory ratio in nonspecific layer II/III cells for amplitude (left), median instantaneous frequency (middle), and frequency (right). There was no significant difference in E/I ratio between mCherry 1.251(0.443) pA/pA, hM4D 1.432(0.854) pA/pA, and hM4D + Saline 1.633(0.738) pA/pA for amplitude (*f* = 1.966; df = 2, 5.47; *p* = 0.173). There was no significant difference in E/I ratio between mCherry 1.693(1.858) Hz/Hz, hM4D 1.427(1.310) Hz/Hz, and hM4D + Saline 2.185(1.269) Hz/Hz for median instantaneous frequency (*f* = 0.770; df = 2, 17.63; *p* = 0.478). There was no significant difference in E/I ratio between mCherry 1.723(1.571) Hz/Hz, hM4D 1.270(1.784) Hz/Hz, and hM4D + Saline 2.123(1.217) Hz/Hz for frequency (*f* = 1.196; df = 2, 16.55; *p* = 0.327). Right panel, Diagram of significant changes in excitation and inhibition after adolescent thalamocortical inhibition. ***b***, Left panel, Schematic of viral injections. Center panel, Excitatory/inhibitory ratio in layer II/III accumbens projecting cortical cells for amplitude (left), median instantaneous frequency (middle), and frequency (right). There was no significant difference in E/I ratio between mCherry 1.316(0.587) pA/pA and hM4D 1.524(0.505) pA/pA for amplitude (*f* = 0.543; df = 1, 6.59; *p* = 0.487). There was no significant difference in E/I ratio between mCherry 1.686(1.368) Hz/Hz and hM4D 1.999(1.715) Hz/Hz for median instantaneous frequency (*f* = 0.836; df = 1, 7.91; *p* = 0.388). There was no significant difference in E/I ratio between mCherry 1.663(0.963) Hz/Hz and hM4D 1.865(1.406) Hz/Hz for frequency (*f* = 0.750; df = 1, 8.10; *p* = 0.411). Right panel, Diagram of significant changes in excitation and inhibition after adolescent thalamocortical inhibition. ***c***, Left panel, Schematic of viral injections. Center panel, Excitatory/inhibitory ratio in layer V/VI thalamic projecting cortical cells for amplitude (left), median instantaneous frequency (middle), and frequency (right). There was no significant difference in E/I ratio between mCherry 1.195(0.500) pA/pA and hM4D 1.376(0.858) pA/pA for amplitude (*f* = 3.625; df = 1; 42; *p* = 0.064). There was no significant difference in E/I ratio between mCherry 1.414(0.485) Hz/Hz and hM4D 1.660(1.143) Hz/Hz for median instantaneous frequency (*f* = 0.975; df = 1, 5.21; *p* = 0.367). There was no significant difference in E/I ratio between mCherry 1.414(0.485) Hz/Hz and hM4D 1.503(1.136) Hz/Hz for frequency (*f* = 1.115; df = 1, 5.31; *p* = 0.337). Right panel, Diagram of significant changes in excitation and inhibition after adolescent thalamocortical inhibition. ***d***, Left panel, Schematic of viral injections. Center panel, Excitatory/inhibitory ratio in layer V/VI accumbens projecting cortical cells for amplitude (left), median instantaneous frequency (middle), and frequency (right). There was no significant difference in E/I ratio between mCherry 1.467(0.624) pA/pA and hM4D 1.730(0.618) pA/pA for amplitude (*f* = 2.220; df = 1, 28; *p* = 0.147). There was no significant difference in E/I ratio between mCherry 2.060(1.840) Hz/Hz and hM4D 2.432(1.435) Hz/Hz for median instantaneous frequency (*f* = 0.412; df = 1, 12.33; *p* = 0.533). There was no significant difference in E/I ratio between mCherry 1.829(1.623) Hz/Hz and hM4D 2.658(1.765) Hz/Hz for frequency (*f* = 0.014; df = 1, 12.10; *p* = 0.908). Right panel, Diagram of significant changes in excitation and inhibition after adolescent thalamocortical inhibition. ***e***, Left panel, Schematic of viral injections. Center panel, Excitatory/inhibitory ratio in cortical projecting cortical cells for amplitude (left), median instantaneous frequency (middle), and frequency (right). There was no significant difference in E/I ratio between mCherry 1.209(0.359) pA/pA and hM4D 1.473(0.403) pA/pA for amplitude (*f* = 1.099; df = 1, 59; *p* = 0.299). There was a significant decrease in E/I ratio between mCherry 2.513(1.915) Hz/Hz and hM4D 1.517(1.670) Hz/Hz for median instantaneous frequency. There was also a significant decrease in E/I ratio between mCherry 2.594(1.607) Hz/Hz and hM4D 1.564(1.544) Hz/Hz for frequency. All reported values are median(interquartile range). Right panel, Diagram of significant changes in excitation and inhibition after adolescent thalamocortical inhibition. *p < 0.05.

In keeping with prior results that found that excitation and inhibition are highly correlated in adolescent rat cortical neurons (Okun and Lampl, 2008), we found a strong correlation in the frequency of excitatory and inhibitory currents in adolescent mice in nonprojection-specific layer II/III cortical neurons (*R*^2^ = 0.524; *p* < 0.001), cortico-accumbens neurons (*R*^2^ = 0.811; *p* < 0.001), corticothalamic (*R*^2^ = 0.850; *p* < 0.001), and corticocortical neurons (*R*^2^ = 0.550; *p* < 0.001). In many cell populations, we observed changes in both excitation and inhibition, suggesting homeostatic mechanisms may compensate for reduced excitatory drive from the thalamus. We therefore calculated the excitation–inhibition balance in each population of neurons identified by layer and projection target (Fig. 6*a–e*). Remarkably, although adolescent thalamic inhibition elicited numerous significant effects on excitatory and inhibitory transmission across cell types and cortical layers, this manipulation did not impact overall excitation–inhibition balance in layer II/III and V/VI cortico-accumbens-projecting neurons and corticothalamic neurons (Fig. 6*a–d*). In contrast, when analyzing the ratio of excitatory to inhibitory currents in contralateral-projecting mPFC neurons, the frequency (*F*_(1,11.37)_ = 5.697; *p* = 0.035) and median of the instantaneous frequency (*F*_(1,9.34)_ = 5.744; *p* = 0.039) of excitatory to inhibitory currents was significantly weighted toward inhibition (Fig. 5*e*).

### P20–P35 thalamocortical inhibition produces cognitive deficits in adult mice

**Figure 7.**
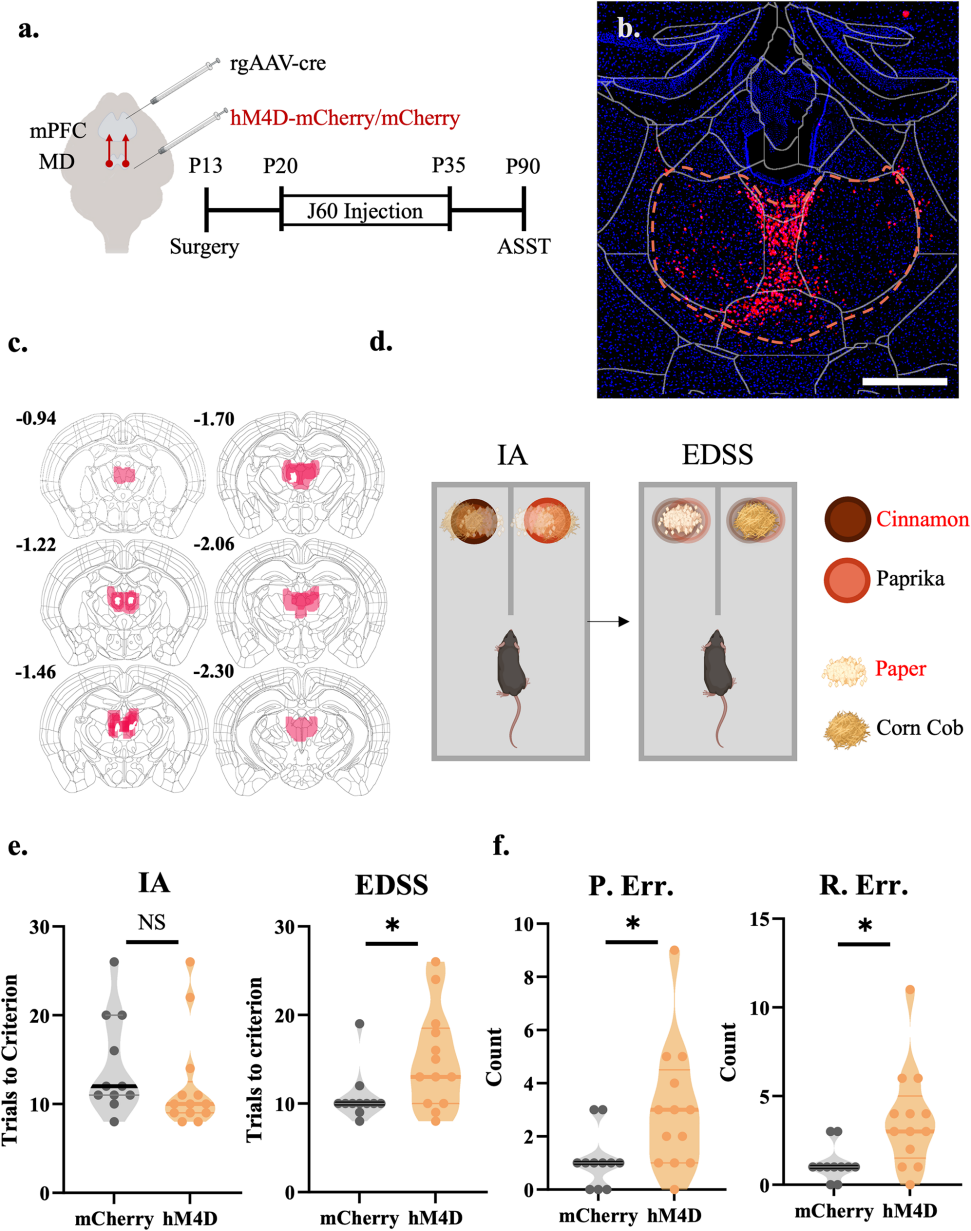
P20–P35 thalamocortical inhibition produces cognitive deficits in adult mice. ***a***, Adolescent experimental timeline and schematic. J60 was administered from P20–P35 to mice expressing hM4D or a control fluorophore (GFP) in the thalamus. Behavioral testing was conducted 55 d later, at P90. Created with https://biorender.com. ***b***, Representative image of hM4D expression in the MD of adult mice. Scale bar, 300 µm. ***c***, Representative viral spread of hM4D receptor in mice that underwent the ASST task. ***d***, Schematic of the ASST. Mice were trained to associate a cinnamon scent with a reward irrespective of bedding medium (IA). After meeting criterion, an EDSS occurred and mice had to associate the paper bedding with a reward irrespective of scent. Red is representative of the rewarded stimulus. ***e***, Adolescent-inhibited hM4D animals are no different than controls in the initial acquisition (IA) of the ASST (left, control: *n* = 11 animals, 14.27 ± 2.276 trials; hM4D, *n* = 13 animals; hM4D, 12.08 ± 2.276 trials; two-sided unpaired *t* test; *t* = 0.9647; df = 22; *p* = 0.3452) but take significantly more trials in the EDSS than controls (right, control: *n* = 11 animals, 10.73 ± 1.871 trials; hM4D, *n* = 13 animals; hM4D, 14.92 ± 1.871 trials; unpaired *t* test; *t* = 2.442; df = 22; *p = 0.0354). ***f***, Adolescent-inhibited hM4D animals committed more perseverative errors (P. Err.) than control animals (left, control: *n* = 11 animals, 1.091 ± 0.7759 trials; hM4D, *n* = 13 animals; hM4D, 3.000 ± 0.7759 trials; unpaired *t* test; *t* = 2.461; df = 22; *p = 0.0222). Perseverative errors are when the mouse continues with the same response strategy following a rule switch. Adolescent-inhibited hM4D animals committed more random errors (R. Err.) than control animals (right, control: *n* = 11 animals, 1.182 ± 0.9010 trials; hM4D, *n* = 13 animals; hM4D, 3.692 ± 0.9010 trials; unpaired *t* test; *t* = 2.786; df = 22; *p = 0.0108) nonperseverative errors are generally considered to be random.

We then determined whether thalamocortical inhibition from P20–P35 has similar long-lasting effects on cognition compared with P20–50 inhibition. J60 was administered from P20 to P35 to mice expressing hM4D or a control fluorophore (GFP) in thalamus-prelimbic cortex-projecting neurons (Fig. 7*a–c*). Behavioral testing was conducted 55 d later, at P90 (Fig. 7*a*). After thalamocortical inhibition, at P90, mice performed the ASST. For initial acquisition, mice were trained to associate a cinnamon scent with a reward irrespective of bedding medium. After meeting criterion, an EDSS occurred, and mice had to associate the paper bedding with a reward irrespective of scent (Fig. 7*d*). Adolescent thalamoprefrontal inhibition did not affect the initial acquisition of the task (*t* = 0.9647; df = 22; *p* = 0.3452) but delayed acquisition of the EDSS compared with controls (*t* = 2.442; df = 22; *p = 0.0354; Fig. 7*e*). Adolescent-inhibited hM4D animals committed more perseverative errors than control animals (*t* = 2.461; df = 22; *p = 0.0222; Fig. 7*f*). Perseverative errors are when the mouse continues with the same response strategy following a set shift. However, adolescent-inhibited hM4D animals also committed more random errors than control animals, and the deficit was not due to a specific error type (*t* = 2.786; df = 22; *p = 0.0108; Fig. 7*f*). These data indicate that a restricted critical window (P20–P35) is sufficient to manifest deficits observed with a longer period of inhibition (P20–P50; Benoit et al., 2022).

## Discussion

To determine thalamocortical activity's importance for prefrontal circuit maturation, we inhibited thalamo-mPFC projections from P20–35 and recorded from prelimbic neurons 24 h later. We made several observations. First, our findings reveal that excitatory transmission to layer II/III is already decreased during adolescence, suggesting that the adult excitation loss (Benoit et al., 2022) does not require maturation to adulthood but arises during adolescence. Second, we measured decreased excitatory and inhibitory transmission to layer II/III NAc- and layer V/VI thalamic-projecting neurons. These inhibitory changes suggest adolescent thalamocortical inhibition leads to alterations in prefrontal connectivity beyond the loss of excitatory thalamic input, previously observed at the anatomical level at P35 (Benoit et al., 2022). Third, we observed a surprising increase in excitatory and inhibitory transmission to layer V/VI NAc-projecting neurons in amplitude but not frequency, suggesting a postsynaptic adaptation.

It is unclear why the amplitude is differentially affected in deep versus superficial layers; however, NAc-projecting neurons in deep layers have distinct molecular characteristics from superficial layers, which may differentially impact their response to adolescent thalamocortical inhibition (Bhattacherjee et al., 2019). Fourth, we measured enhanced inhibition in corticocortical connectivity, providing further evidence for an intrinsic adaptation in response to adolescent thalamocortical inhibition. Intriguingly, while changes in excitatory and/or inhibitory drive were noted in subcortical projection neurons, a change in the overall E/I balance was only measured in cross-hemispheric mPFC projections and not in the three subcortical projections analyzed. As functional stability in neural circuits can be sustained by homeostatic rebalancing of E/I inputs, the largest functional effect of adolescent thalamocortical inhibition on prefrontal circuitry may be a decrease in the activity of callosal-projecting mPFC pyramidal neurons. Decreased activity of callosal-projecting neurons during adolescence could further impact subsequent mPFC maturation and adult function. Finally, we established that this narrowed window of inhibition (P20–P35) is sufficient to produce cognitive deficits in the adult mouse.

### Decreased excitatory and inhibitory transmission in the mPFC arises during adolescence

Previous work demonstrated that adolescent thalamocortical inhibition (P20–P50) resulted in cognitive and physiological deficits in the adult mouse, including a loss of excitatory transmission in nonprojection-specific layer II/III pyramidal neurons. Here, we observe that loss of excitatory inputs onto this cell population is present immediately following adolescent inhibition. The similarity between the findings at P35 and in adulthood suggests that changes in this population develop early and then persist into adulthood. This also suggests that P20–35 may be an important time window when connections onto layer II/III cells are refined based on typical developmental activity. Regarding which excitatory inputs onto layer II/III neurons are reduced by thalamocortical inhibition, retrograde tracing studies suggest MD inputs are lost both in adulthood and at P35 (Benoit et al, 2022). Whether any of the other changes that we measured in adolescence, such as enhanced inhibition to callosal-projecting layer II/III neurons, are present in adulthood remains to be shown.

Regarding these other changes, we found that excitatory and inhibitory currents were both decreased in layer II/III NAc-projecting and layer V/VI MD-projecting pyramidal neurons. The effect on NAc-projecting neurons demonstrates an impact on connections beyond those linking the thalamus and cortex. The significant loss of inhibitory transmission on these neurons could result from a reduction in thalamic projections onto interneurons (Yang et al., 2021). The MD directly innervates layer II/III parvalbumin (PV+) and other interneurons, providing a source of feedforward inhibition to cortical-projecting neurons (Delevich et al., 2015; Canetta et al., 2020). Changes in interneuron-driven inhibition in NAc/MD-projecting neurons measured in the slice could reflect either reduced excitatory drive onto PV+ (or other) interneurons or adaptations within prelimbic interneurons following thalamic inhibition. The loss of excitatory inputs to layer V/VI pyramidal neurons, which are only believed to receive weak direct MD input (Collins et al., 2018), suggests excitatory inputs other than those of the MD may be compromised following adolescent thalamic inhibition. Whether these changes persist into adulthood remains an interesting, yet unanswered, question.

### Deeper accumbens-projecting neurons show postsynaptic adaptations to MD inhibition

Surprisingly, we found that the impact of adolescent thalamic inhibition on excitatory and inhibitory inputs was not the same for all mPFC cell types. For example, in layer V/VI NAc-projecting neurons, there was no change in the frequency of excitatory or inhibitory currents although the amplitude of both increased. Changes in amplitude are thought to reflect postsynaptic alterations; however, it is not clear for what the increased amplitude observed in these cells would be compensating. Different effects on this neuronal population may reflect differences in the relative levels of direct MD input, which has not been measured for layer V/VI NAc-projecting neurons.

### Adolescent thalamocortical inhibition increases inhibitory inputs to callosal-projecting neurons

In contrast to subcortical layer II/III NAc- and layer V/VI MD-projecting neurons, the frequency of excitatory inputs to callosal-projecting layer II/III mPFC neurons did not change, and inhibitory inputs to this population were increased. The absence of excitatory transmission changes in this population is particularly surprising, given they receive strong MD inputs (Anastasiades et al., 2018). Why excitatory inputs to this population are spared remains unknown but suggests that excitatory inputs from other brain regions may compensate for reduced thalamocortical input. The origin of the increase in inhibition in callosal-projecting neurons following adolescent thalamocortical inhibition is unclear but may involve disinhibition via somatostatin (SST+), vasoactive intestinal polypeptide (VIP+), and PV+ interneurons. Consistent with this, both VIP+ and SST+ interneurons receive MD input and inhibit other interneurons including PV+ interneurons (Ahrlund-Richter et al., 2019; Sun et al., 2019; Canetta et al., 2020).

The increase in inhibitory transmission is particularly surprising given that inhibition was decreased in NAc-projecting neurons found in the same cortical layers. The reason for the specificity toward intracortical connectivity is unclear but some interneurons specifically target callosal-projecting pyramidal neurons (Pi et al., 2013; Cho et al., 2023). The observation that adolescent thalamic inhibition results in opposing effects on inhibitory tone onto NAc-projecting versus callosal-projecting layer II/III neurons may explain why recordings from nonprojection-specific neurons show no significant differences in inhibitory tone. In fact, we see a wide distribution of sIPSC frequencies across neurons depicted in Figure 1*h*, which could result from some neurons having decreased sIPSC frequencies in response to adolescent thalamic inhibition while other neurons may have increased frequency.

### Adolescent thalamic inhibition only affects E/I balance in layer II/III callosal-projecting pyramidal neurons

Overall, the E/I ratio following adolescent thalamocortical inhibition displays remarkable homeostatic stability. Adolescent thalamocortical inhibition did not impact E/I balance in any cell population studied except for layer II/III callosal-projecting neurons, which showed a significant reduction in the sEPSC/IPSC frequency ratio. Thus, callosal-projecting neurons may not undergo homeostatic adaptation compared with subcortically projecting neurons. Whether the effect on E/I balance in callosal-projecting neurons is sustained into adulthood is not yet known. Based on changes in E/I balance in adolescence, cortically projecting neurons might experience the greatest change in net drive immediately following adolescent thalamocortical inhibition, with the overall effect of being less active in vivo. This change may in turn contribute to a loss of cross-hemispheric cortical communication, hypothesized to be important for cognitive processes (Hasegawa et al., 1998; Holler-Wallscheid et al., 2017). These effects may in turn depress cortical output to other neural circuits, resulting in more widespread effects on prefrontal network function. These possibilities remain to be tested.

### Possible mechanisms of mPFC plasticity induced by adolescent thalamocortical inhibition

Recent studies also point to intracortical mechanisms mediating maturation of cortical circuitry. Anterior cingulate (ACC) neurons show greater excitatory synaptic inputs during adolescence than adulthood and inhibition of ACC to visual cortex projection neurons during adolescence disrupts the maintenance of local connectivity within the ACC (Nabel et al., 2020). By analogy, if adolescent thalamocortical inhibition decreases activity of MD- or NAc-projecting mPFC neurons during adolescence, this may lead to a disruption in the maintenance of local excitation in the mPFC. A different mechanism has been described within the visual cortex. Here, inhibition of layer II/III neurons during the critical period of primary visual cortex development (P24–29) led to excitatory synaptic scaling and increased intrinsic excitability suggesting homeostatic plasticity as a mechanism affecting visual cortex maturation (Wen and Turrigiano, 2021). Further studies on synaptic scaling could identify the plasticity mechanisms in mPFC circuitry induced by adolescent thalamocortical inhibition.

### A narrowed window of adolescent thalamocortical inhibition regulates adult cognition

Adolescent thalamocortical inhibition from P20–35 elicited similar deficits in attentional set shifting in the adult mouse at P90 as inhibition in a broader time window from P20 to P50 (Benoit et al., 2022), narrowing the sensitive window for this effect. While we cannot exclude that thalamocortical inhibition in a different developmental window might also impact prefrontal connectivity and behavior, our current findings implicate early adolescence as a sensitive period during which thalamic input regulates prefrontal cortex development. Moreover, while the dual virus approach targets midline and mediodorsal thalamic projections to the mPFC, some of these neurons, especially in the paraventricular, have been shown to possess collaterals to the nucleus accumbens, bed nucleus of the stria terminalis, and the amygdala (Dong et al., 2017). It is possible that long-term changes in brain function due to inhibition of these collateral projections contribute to the behavioral deficits observed in the adult animal.

### Conclusions

In sum, our data demonstrate that adolescent thalamocortical inhibition results in projection-specific changes in synaptic connectivity in the mPFC that become evident already during adolescence. The dysregulation of multiple neural circuits demonstrates a widespread effect of adolescent thalamocortical inhibition that impacts more than just thalamocortical circuitry and deficits cannot be explained solely by a lack of anatomical input. Rather, they generate widespread adaptative and possibly maladaptive physiological responses within prefrontal circuitry.

### Future directions

Future studies using optogenetic approaches to probe functional connectivity between specific excitatory or inhibitory inputs to mPFC pyramidal neurons will be able to address the synaptic origin of these complex adaptations. Bath application of J60 of adult rats expressing hM4DGi in mPFC terminals of the thalamus has been shown to reduce sEPSCs by ∼30% (Ferguson and Gao, 2018). Probing this in adolescent thalamic inhibited mice will allow us to determine the thalamic contribution to the observed changes in synaptic connectivity. Furthermore, it will be important to explore how these physiological changes will scale into adulthood. If they do, it is likely adolescent thalamocortical inhibition would lead to changes in prefrontal circuitry that are “hardwired” into adulthood. Alternatively, changes identified in adolescence may be transient, setting in motion subsequent processes that alter adult prefrontal function. Finally, future research will address whether disinhibition of callosal cortical-projecting neurons during adolescence following adolescent thalamocortical inhibition will rescue long-term negative effects and determine whether adolescence is a potential target for therapeutic intervention.

## Synthesis

Reviewing Editor: Fabienne Poulain, University of South Carolina Decisions are customarily a result of the Reviewing Editor and the peer reviewers coming together and discussing their recommendations until a consensus is reached. When revisions are invited, a fact-based synthesis statement explaining their decision and outlining what is needed to prepare a revision will be listed below. The following reviewer(s) agreed to reveal their identity: NONE. Note: If this manuscript was transferred from JNeurosci and a decision was made to accept the manuscript without peer review, a brief statement to this effect will instead be what is listed below.

The authors effectively addressed all the issues raised by the reviewers. Their study is innovative, and the findings clearly advance knowledge regarding the spatiotemporal impact that thalamocortical projections play in the development of the medial prefrontal cortex circuit during adolescence.
